# Reliable Detection of Implicit Waveform-Specific Learning in Continuous Tracking Task Paradigm

**DOI:** 10.1038/s41598-017-11977-5

**Published:** 2017-09-26

**Authors:** Limin Yang, Feng Wan, Wenya Nan, Frank Zhu, Yong Hu

**Affiliations:** 1 Department of Electrical and Computer Engineering, University of Macau, Macau S.A.R., China; 2 Department of Psychology, College of Education, Shanghai Normal University, Shanghai, China; 3 Faculty of Education, The University of Hong Kong, Hong Kong S.A.R., China; 4 Department of Surgery, Li Ka Shing Faculty of Medicine, The University of Hong Kong, Hong Kong S.A.R., China; 5 Department of Orthopaedics and Traumatology, Li Ka Shing Faculty of Medicine, The University of Hong Kong, Hong Kong S.A.R., China

## Abstract

Implicit waveform-specific (IWS) learning during a typical continuous tracking task paradigm has been reported for decades, as evidenced by better tracking improvement on the repeated segment of a specific target waveform than random segments. However, the occurrence of the IWS learning in such a task paradigm has been challenged by several unsuccessful results in recent literature. This research concerns reliable detection of the induced IWS learning and to this end, proposes to use the similarity between the cursor and the target along the direction corresponding to the waveform pattern as the performance measure. A 3-day experiment designed with full examination on IWS learning including a practice phase, an immediate test phase and a consolidation test phase after 24 hours was conducted to validate the feasibility and sensitivity of the Pearson’s correlation coefficient on the vertical movement *r*_*v*_ in this study. Experiment results indicate that *r*_*v*_ is more sensitive in detecting the IWS learning in all phases compared to the conventional root mean square error (RMSE) performance measure. The findings confirm the importance of the performance measure in implicit learning research and the similarity measure in accordance with the waveform could be promising for waveform-specific learning detection in this paradigm.

## Introduction

Implicit learning is generally defined as a natural learning process, in which individual devotes sufficient attention to a structured stimulus environment without a clear awareness of what to learn or any conscious operation such as explicit strategies for learning^1^. The “implicit learning” phenomenon has been elicited in many learning processes of fundamental abilities in experiment situations, including language acquisition, object knowledge formation and motor learning^2^. Specifically in motor skills domain, the implicit learning involving many motor components is termed as *implicit motor learning* ^3^, which has been investigated by a good number of studies^4–8^ using the continuous tracking task paradigm developed by Pew^9^.

In a typical continuous tracking task, implicit motor learning is represented by implicit waveform-specific (IWS) learning. The participants are instructed to track a target horizontally moving across the screen following an invisible trajectory with a hand-driven device. The waveform of the trajectory normally consists of three equal-duration segments, in which the middle segment is repeated throughout all trials while the other two segments are randomly generated for each trial under complexity control^10^. When participant’s continuous tracking performance on the middle repeated segment outperforms the outer random segments, waveform-specific learning happens. Generally, the participant is blinded to the segment composition and unaware of the existence of the repeated segment, which enables one to conclude that the waveform-specific learning is implicit.

The occurrence of IWS learning in the continuous tracking task paradigm was firstly reported by Pew^9^. Since then, many researchers utilized the continuous tracking task paradigm to investigate implicit motor learning in different research contexts such as comparison of implicit and explicit learning^10,11^, validation on the occurrence of implicit learning^4,11,12^, examination on the capability of implicit learning in older and younger adults^13^ and patients^14,15^, and investigation on oculomotor and manual coordination in implicit motor learning^16^.

Although most of the studies reported the occurrence of IWS learning in the continuous tracking task paradigm^4,5,9–13,16,17^, the failure to observe the IWS learning in several studies challenged the reliability of this task paradigm. Chambaron *et al*.^18^ observed IWS learning only when using exactly the same repeated segment reported by Wulf *et al*.^12^, but failed when the repeated waveform patterns assigned to the participants varied from each other. They inferred that the superior tracking performance in repeated segment observed by Wulf *et al*.^12^ might be due to the easiness of the repeated segment. However, such an inference was debatable given the procedural difference (e.g. numbers of practice trials, task speed and hand-driven device) between these two studies: it was pointed out that in contrast to a larger amount of practice in the original experiment^12^, only a single practice session of 12 trials in the replication^18^ might be insufficient for any effect of practice to occur^4^. In another study^19^, Lang *et al*. did not observe IWS learning in a standard continuous tracking task and they attributed the failure to the ceiling effect of tracking performance. This presumption was consistent with the viewpoint that the IWS learning did occur but the expression of knowledge was suffering from a ceiling effect^4^. In contrast to these negative results^18,19^, the validation study^4^ and our recent work^5^ successfully demonstrated the IWS learning in the continuous tracking task, in the condition that the repeated segments assigned to each participant were different.

A number of researchers have attempted to improve the continuous tracking task paradigm from different aspects, in order to increase its reliability for implicit motor learning study^3–5,19^. A major effort has been devoted to reinforcing the IWS learning effect during the task, such as Lang *et al*.’s investigations on enhancing the implicit learning through a better predictability by increasing target sequence regularities in the repeated segments^3^, and removing the negative guidance effect that prevents the participants from learning by suppressing visual feedback^19^. Another approach concentrates on the detection of the induced IWS learning, for instance Künzell *et al*. concerned how the tracking path characteristics as well as the target speed affected the IWS learning detection^4^, and our previous work tested the time-on-task effect on the detection and also provided refinements on the paradigm for more effective detection^5^.

This paper argues that a reliable tracking performance measure with specific sensitivity to IWS learning is critical to the continuous tracking task paradigm. The discrepant results from the aforementioned studies, to some extent, revealed the importance and difficulty of how to detect the IWS learning reliably and effectively. Currently, the root mean square error (RMSE) in screen pixels is the most widely adopted performance measure in the continuous tracking task for implicit motor learning research^5,6,8,9,12,20,21^. However, the RMSE index simply sums up the squared errors of every tracking, which focuses more on local errors than global similarity and is vulnerable to accidental errors that are unrelated to IWS learning, for instance the mistakes caused by hand-driven device control. Some researchers also used other conceptually similar dependent variables as performance measure, such as the integrated absolute error^9^ and the radial error^22^, which also concerned much detailed local information. The low sensitivity and specificity of these existing performance measures in the IWS learning detection may imperil the reliability of the continuous tracking task paradigm for implicit learning studies. Differently, Lang *et al*.^19^ applied the inter-correlation coefficient on the horizontal movement, which is essentially a typical similarity indicator of two time series calculated by Pearson’s correlation test, to assess the tracking performance. Unfortunately, they still failed in detecting the IWS learning in the traditional continuous tracking task^19^, which might result from the ceiling effect due to the inappropriate difficulty set for the tracking task in the designed experiment as stated therein^19^.

Aiming at reliable detection of IWS learning in the continuous tracking task paradigm, this study investigated the feasibility and sensitivity of the Pearson’s correlation coefficient on the vertical movement (denoted by *r*_*v*_) as the performance measure, in comparison with the conventional measure using RMSE. The experiment was particularly designed with a practice phase, an immediate test phase and a consolidation test phase, for a full examination on IWS learning. More specifically, twenty-four participants performed the continuous tracking task on three days (i.e. Day 1, Day 2 and Day 3) in which they were instructed to track a moving target displayed on a monitor with a stylus and pen tablet. There was one block on Day 1 as the performance baseline. On Day 2, five consecutive practice blocks followed by one transfer block and one retention block were performed. On Day 3, two retention blocks with one transfer block in-between were constructed for the consolidation test. Each block consisted of four trials and each trial was divided into three segments. In all trials, the waveforms in the first segment (Seg1) and the third segment (Seg3) were randomly generated, in the middle segment (Seg2) the waveform was repeated over trials in the practice and retention blocks, and randomly generated in the baseline and transfer blocks.

## Results

**Figure 1:**
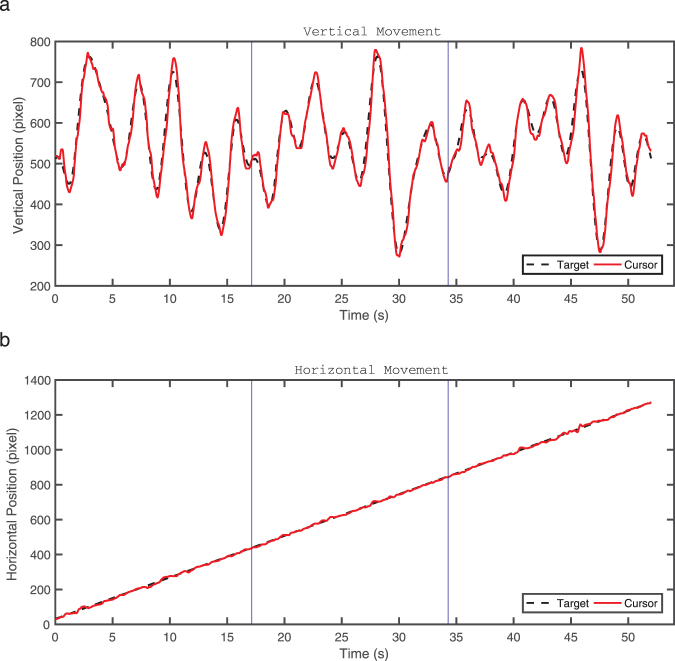
The decomposition of tracking movements in a single continuous tracking trial. (**a**) Vertical movements of the tracked target and controlled cursor. (**b**) Horizontal movements of the tracked target and the controlled cursor. (Blue lines are the boundaries between two consecutive segments).

**Figure 2:**
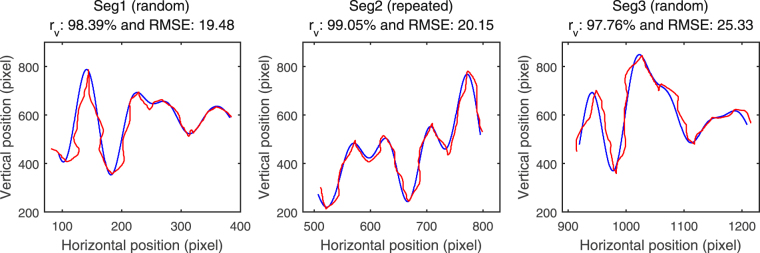
The target movement (blue curves) and the cursor movement (red curves) in three segments of an example trial. (*Random: with random waveform patterns. Repeated: with repeated waveform patterns*).

The illustration of the continuous tracking trajectories with horizontal movements and vertical movements is presented in Fig. 1. Regarding the tracking performance measures, a smaller RMSE or a higher *r*_*v*_ indicates a better tracking performance. Figure 2 gives an example of the target and the cursor movements of three segments in one trial to show the individual tracking performance. In order to fully examine the IWS learning in the continuous tracking task, two-way analysis of variance (ANOVA) with repeated measures were performed for the tracking performance measures. Both tracking performance measures were normally distributed examined by Shapiro-Wilk test, and Greenhouse-Geisser adjustments were used if Mauchley’s test showed that assumptions of sphericity were violated. The analyses and comparisons between RMSE and *r*_*v*_ were performed in three aspects based on the time course, including (1) across the practice phase, (2) during the immediate test phase on Day 2, and (3) during the consolidation test phase on Day 3, which are depicted in the following paragraphs.

### Across the practice phase

**Figure 3:**
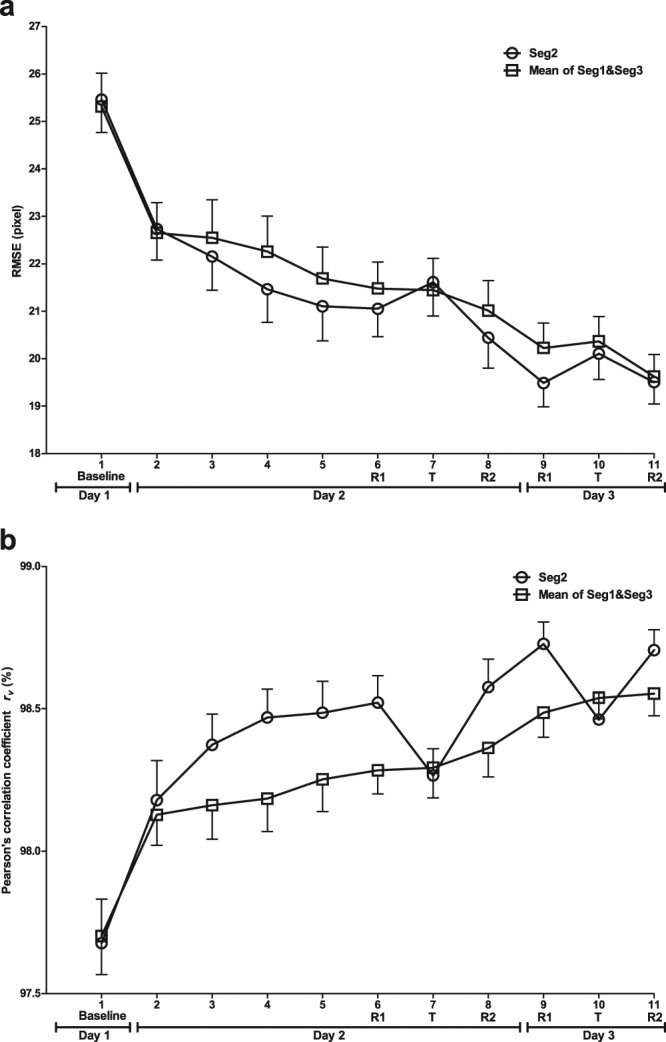
Mean tracking performance across the 3-day experiment for Seg2 and the mean of Seg1 and Seg3. R1 and R2 represent the retention tests 1 and 2 respectively with Seg2 as repeated segment while T represents the transfer test with Seg2 as random segment. (**a**) Measured by RMSE. (**b**) Measured by Pearson’s correlation coefficient *r*_*v*_.

Tracking performance measured by RMSE and *r*_*v*_ on the Seg2 and the mean of random segments Seg1 and Seg3 across the practice phase were analyzed respectively by 2 (Segment: Seg2 and the average of Seg1 and Seg3) × 6 (Block: Block 1 to Block 6) repeated ANOVA. Regarding both RMSE and *r*_*v*_, a main effect of Block was evident (RMSE: *F*_2.64, 60.65_ = 28.009, *p* < 0.0001, *partial*-*η* ^2^ = 0.549; *r*_*v*_: *F*_1.91, 49.96_ = 22.622, *p* < 0.0001, *partial*-*η* ^2^ = 0.496), indicating the improvement of tracking performance by practice. This is consistent with the tracking performance curve in Fig. 3 where all segments showed a decreasing trend in RMSE and an increasing trend in *r*_*v*_ across the practice blocks.

However, neither a main effect of Segment (*F*_1, 23_ = 2.245, *p* = 0.148, *partial*-*η* ^2^ = 0.089) nor a Segment × Block interaction (*F*_3.33, 76.55_ = 1.84, *p* = 0.141, *partial*-*η* ^2^ = 0.074) was evident for RMSE, indicating that the improvement of tracking performance over blocks measured by RMSE had no significant difference between the Seg2 and the mean of random segments Seg1 and Seg3. On the contrary, Segment showed a significant main effect on *r*_*v*_ (*F*_1, 23_ = 5.348, *p* = 0.03, *partial*-*η* ^2^ = 0.189) so that the tracking performance in the Seg2 measured by *r*_*v*_ was superior to the mean of random segments Seg1 and Seg3. Importantly, an expected significant Segment × Block interaction (*F*_3.06, 70.44_ = 2.965, *p* = 0.037, *partial*-*η* ^2^ = 0.114) was observed for *r*_*v*_, suggesting that the improvement of tracking performance over blocks in the Seg2 was more significant than that of the random segments Seg1 and Seg3. This result provided evidence of IWS learning in the practice phase.

### During the immediate test phase

**Figure 4:**
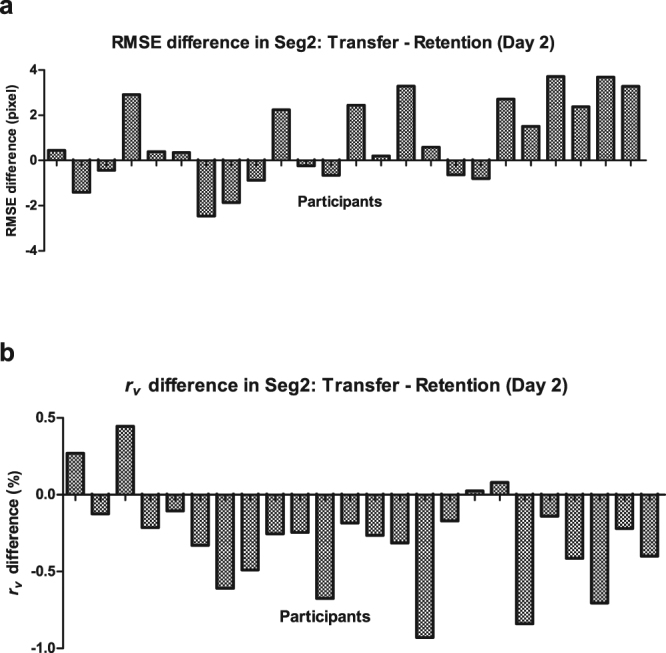
The tracking performance difference in Seg2 between transfer and retention tests on Day 2 measured by (**a**). RMSE; (**b**) Pearson’s correlation coefficient *r*_*v*_.

The immediate test phase on Day 2 included the transfer test in Block 7 and the retention test which was adjusted to be the average of Block 6 and Block 8 for counterbalance. Figure 4 presents the tracking performance difference between the retention test and the transfer test (i.e. Transfer - Retention) across subjects (*n* = 24) measured by both RMSE and *r*_*v*_. It can be observed that the IWS learning was detected in most participants by both measures; nevertheless, the tracking performance measured by *r*_*v*_ led to detection of the IWS learning in more participants than that measured by RMSE. Moreover, tracking performance measured by RMSE and *r*_*v*_ were analyzed respectively by a 2 (Segment: Seg2 and the average of Seg1 and Seg3) × 2 (Test: transfer test and retention test) repeated ANOVA.

For RMSE, a main effect of Test (*F*_1, 23_ = 4.804, *p* = 0.039, *partial*-*η* ^2^ = 0.173) was observed, suggesting that the tracking performance in the transfer test was significantly lower than that in the retention test. However, neither a main effect of Segment (*F*_1, 23_ = 0.614, *p* = 0.441, *partial*-*η* ^2^ = 0.026) nor a Segment × Test interaction (*F*_1, 23_ = 2.894, *p* = 0.102, *partial*-*η* ^2^ = 0.112) was found, indicating that the decrease of tracking performance from the retention test to the transfer test had no significant difference between the Seg2 and the random segments Seg1 and Seg3.

For *r*_*v*_, there was a significant main effect of Test, with tracking performance decreasing from the retention test to the transfer test (*F*_1, 23_ = 12.612, *p* = 0.002, *partial*-*η* ^2^ = 0.354), and a significant main effect of Segment with higher tracking performance in the Seg2 in comparison with the random segments Seg1 and Seg3 (*F*_1, 23_ = 6.141, *p* = 0.021, *partial*-*η* ^2^ = 0.211). More in *r*_*v*_ (*F*_1, 23_ = 11.659, *p* = 0.002, *partial*-*η* ^2^ = 0.336), indicating that the decrease of tracking performance from the retention test to the transfer test in the Seg2 was significant larger than that in the random segments Seg1 and Seg3. This finding was consistent with our expectation and supported the occurrence of the IWS learning.

### During the consolidation test phase

In order to compare the detection sensitivity of two performance measures to the offline consolidation of IWS learning, the tracking performance measured by RMSE and *r*_*v*_ in the consolidation test phase on Day 3 were also analyzed respectively by a 2 (Segment: Seg2 and the average of Seg1 and Seg3) × 2 (Test: transfer test and retention test) repeated ANOVA.

For RMSE, the main effect of Test (*F*_1, 23_ = 5.358, *p* = 0.03, *partial*-*η* ^2^ = 0.189) was significant and the main effect of Segment approached significance (*F*_1, 23_ = 3.578, *p* = 0.071, *partial*-*η* ^2^ = 0.135). Nevertheless, the Segment × Test interaction (*F*_1, 23_ = 0.158, *p* = 0.695, *partial*-*η* ^2^ = 0.007) was far from evident. These results suggested that although the tracking performance significantly decreased from the retention test to the transfer test and the tracking performance was lower in the random segments Seg1 and Seg3 than the Seg2, the decrease of tracking performance did not have difference between the Seg2 and the random segments Seg1 and Seg3.

For *r*_*v*_, there was a significant main effect of Test (*F*_1, 23_ = 4.515, *p* = 0.045, *partial*-*η* ^2^ = 0.164) but no main effect of Segment (*F*_1, 23_ = 1.846, *p* = 0.187, *partial*-*η* ^2^ = 0.074). Nonetheless, we observed a significant Segment × Test interaction (*F*_1, 23_ = 9.765, *p* = 0.005, *partial*-*η* ^2^ = 0.298), implying that the decrease of tracking performance was more pronounced in the Seg2 than in the random segments Seg1 and Seg3 when the repeated waveform pattern of Seg2 in the retention test was replaced by a random pattern in the transfer test. This result demonstrated the offline consolidation of IWS learning.

## Discussion

In this study, we proposed to use the performance measure, i.e., the Pearson’s correlation coefficient on the vertical movement *r*_*v*_, to detect the IWS learning in the continuous tracking task paradigm. The proposed measure has been investigated through a carefully designed experiment which comprises a practice phase, an immediate test phase and a consolidation test phase after 24 hours. To the best of our knowledge, it is the first time to investigate IWS learning in all three phases within one experiment, which enabled a full examination on IWS learning in all different phases and ensured the reliability of the experiment results. The feasibility and sensitivity of *r*_*v*_ on the detection of IWS learning was compared with the conventional RMSE measure that is widely used for the continuous tracking performance and the experiment results indicated that *r*_*v*_ was superior to RMSE in the detection of IWS learning in the continuous tracking task paradigm.

In the practice phase, the ANOVA results showed a significant main effect of Block on both RMSE and *r*_*v*_, indicating that the tracking performance can be significantly improved by practice. This result is in line with our expectation and consistent with our previous work^5^ and the validation study of continuous tracking task for implicit motor learning^4^. More importantly, the Segment × Block interaction revealed significance in *r*_*v*_, indicating that the middle segment showed significant larger improvement over practice than the outer random segments, which provided a strong evidence of IWS learning. On the contrary, no Segment × Block interaction was found in RMSE. Künzell *et al*.^4^ and our previous work^5^ did not find the IWS learning effect in the practice phase using RMSE as the performance measure, either. A possible reason suggested by Künzell *et al*.^4^ on the lack of IWS learning detected during practice says that, although the IWS learning did happen, the expression of learning effect might suffer from a ceiling effect. The analyses and comparisons between RMSE and *r*_*v*_ in this study provided another possibility: the RMSE for detecting IWS learning in these two aforementioned papers might be not sensitive enough to reflect the true extent of the learning effect. Also, the experiment results demonstrated that *r*_*v*_ possesses a higher sensitivity than RMSE to reflect the IWS learning in the practice phase and in the immediate test phase.

As one of the typical tasks to induce implicit motor learning, the continuous tracking task typically involves two types of learning: general motor skill (GMS) learning and IWS learning. GMS learning refers to the acquisition of expertise with the general requirement of the task^23^, and it occurs when tracking both the random and repeated segments. As no waveform-specific learning occurs in the random segments, GMS learning can be measured by the tracking performance improvement in the random segments across practice blocks^23^. IWS learning is a specific representation of implicit learning in the repeated segment, and therefore it can be seen that GMS learning and IWS learning happen simultaneously in the repeated segment across the blocks. Both the GMS learning and IWS learning contribute to the tracking accuracy in the calculation of the performance measure, which may increase the difficulty of the IWS learning detection.

Obviously, in this task paradigm what we really aimed to measure is the waveform-specific learning performance, which concerns how similar the trajectory drawn by the participant is to the given waveform pattern. RMSE squares the point-to-point distance errors and does not count the direction of errors, which may cause ambiguity about of the exact cursor position. Moreover, RMSE summarizes the squared errors at each point but not evaluates the shape of the whole cursor trajectory in comparison with the target trajectory that we care the most. In addition, RMSE is easy to be contaminated by accidental errors that are unrelated to IWS learning, which may hinder or even overwhelm the reflection of IWS learning. On the contrary, as a typical similarity indicator, Pearson’s correlation coefficient measures the degree of resemblance between two trajectories, which conceptually differs from RMSE and is closer to what we aimed to measure. For example, in Fig. 2 it can been seen that the tracking performance in the repeated segment Seg2 is better than that in the random segment Seg1, which can be reflected by *r*_*v*_ rather than RMSE. What is more, the *r*_*v*_ in this study counts less GMS learning effect than RMSE, as the *r*_*v*_ focuses more on the IWS learning induced by the waveforms specifically designed in the vertical direction. As shown in Fig. 1, the waveform pattern of the target movements consists of a combination of a sinusoidal trajectory in the vertical direction and a uniform rectilinear motion in the horizontal direction. These two different types of movements in two orthogonal directions are independent, implying that only the vertical movements carry the information of the repeated waveform pattern while the horizontal movements do not. As illustrated in Fig. 1b, no waveform-specific information is contained in the horizontal direction, and thus the horizontal tracking errors mainly arise from GMS learning but not IWS learning. Consequently, the Pearson’s correlation coefficient *r*_*v*_ that concentrates on the vertical movements can reduce the interference of GMS learning especially from the horizontal direction and consequently measures IWS learning more precisely.

In addition to the implicit motor learning detection, performance measure is also a vital factor for the detection of implicit motor learning consolidation. Offline consolidation is an important issue for motor learning investigation in the continuous tracking task paradigm. Consolidation refers to that performance is robust and resistant to decay and interference with time passing and without further practice^24,25^. It can be assessed by repeating the test phase of the task separated by a period of time in which participants are not concerned with the task. Therefore, IWS learning consolidation can be measured by repeating the immediate test phase on a second day^5^. In this study, during the consolidation test phase on Day 3, it can be observed from Fig. 3 that RMSE showed an expected increase and *r*_*v*_ had an expected decrease in the middle segment Seg2 in the transfer test. However, although RMSE detected the tracking performance difference between the transfer test and the retention test, it failed to reach significance in evaluating the tracking performance drop in the Seg2 from the retention test to the transfer test. Different from RMSE, *r*_*v*_ presented a significantly sharper decline in the transfer test as a typical evidence of the IWS learning consolidation occurrence. These statistical results revealed that the offline consolidation of IWS learning can be significantly reflected by *r*_*v*_ rather than RMSE, which indicated that *r*_*v*_ also outperformed RMSE in the detection of IWS learning consolidation. These findings further underlined the importance of appropriate performance measure to reflect the true extent of implicit motor learning.

A lot of efforts have been put in previous studies in order to increase the reliability for implicit motor learning research using the continuous tracking task paradigm, while on the other hand this reliability is reflected mainly by whether and how successful the IWS learning can be detected. As the GMS learning and the IWS learning may occur simultaneously, a successful detection of IWS learning depends on not only how strong the IWS learning is induced, but also how sensitive to the IWS learning the performance measure is, especially under the interference of GMS learning and other factors. This study proposed to use similarity in the waveform direction as the performance measure and the experiment results demonstrated that the proposed measure *r*_*v*_ is superior to the widely used performance measure RMSE, leading to successful detecting of IWS learning in all three phases. This revealed the importance of the performance measure in the detection of IWS learning and provided more confidence when applying this paradigm as a tool for implicit motor learning research.

## Method

### Participant

A total of twenty-four right-handed young volunteers aged from 19 to 31 (mean: 24.1, *SD*: 3.06, 15 male and 9 female) participated in this study. All participants had normal or corrected-to-normal vision and none of them had prior experience or knowledge of the continuous tracking task. Informed consents were signed before the experiment and honorarium for participation (approximately US $20) were paid after completing the whole experiment for all participants. This experiment was in accordance with the Declaration of Helsinki and approved by the Research Ethics Committee (University of Macau).

### Task

Participant was seated comfortably in front of an LCD monitor (Sony, 17-inch, 1280 $\times$ 1024 pixel resolution) at a typical viewing distance of around 60 cm. The area of full screen was proportionally projected to a large pen tablet (PTH-851, Wacom Intuos pro, Japan) with an active area of 12.8 $\times$ 8.0 inch. Holding a stylus with their right hand, participants were instructed to control the movement of a cross-shaped white cursor to track a red dot with a diameter of 9 mm displayed on the screen. The ratio of the pen movement on the tablet and the cursor movement on the monitor was calibrated to reach exactly 3:4. The goal of this task was to track a targeted red dot moving horizontally with an invisible sinusoidal trajectory. A custom Java program (Sun Microsystems, Santa Clara, CA) was applied to generate the waveform patterns and present the movements of the target and cursor. In the meantime, both the trajectories of targeted red dot and manually controlled cursor were recorded by this program at a sampling rate of 32 Hz. The horizontal and vertical movements of the trajectories were respectively recorded in (*x*, *y*) coordinates in time series.

One trial composed of three segments with equal duration. In each segment, the movement of the targeted red dot consisted of the movements in both the horizontal and vertical directions. The horizontal movement was a uniform rectilinear motion, while the vertical movement was specified by the waveform as shown in equation (1) which was generated by sampling from a sine-cosine series.

1 $${\alpha }_{i}={b}_{0}+{a}_{1}\,\sin \,{\theta }_{i}+{b}_{1}\,\cos \,{\theta }_{i}+{a}_{2}\,\sin \,2{\theta }_{i}+{b}_{2}\,\cos \,2{\theta }_{i}+{a}_{3}\,\sin \,3{\theta }_{i}+{b}_{3}\,\cos \,3{\theta }_{i}+{a}_{4}\,\sin \,4{\theta }_{i}+{b}_{4}\,\cos \,4{\theta }_{i}+{a}_{5}\,\sin \,5{\theta }_{i}+{b}_{5}\,\cos \,5{\theta }_{i}+{a}_{6}\,\sin \,6{\theta }_{i}+{b}_{6}\,\cos \,6{\theta }_{i}$$

${\alpha }_{i}$ is the rounded vertical coordinate of the *i*-th position at which the target is to be displayed, ${\theta }_{i}=i\times 2.14\pi /(time\times freq)$, with *time* representing the segment duration and *freq* representing the sampling frequency. The duration is 17.14 s, and the sampling frequency for display is chosen as the same as that for recording for consistency and simplicity. In order to create a smooth transition between segments, the first 15% and last 15% of each segment was transformed to ensure that the initial and final locations of each segment fell on the horizontal line in the middle of the screen. Therefore, only the rest 70% of each segment (i.e., 1.5$\pi$ out of $2.14\pi$ or 12 seconds in duration) was under complexity control and subsequently analyzed for tracking performance evaluation. Each of the three segments (Seg1, Seg2 and Seg3) had its own waveform pattern. The coefficients of the waveform patterns for all the three segments were generated following two criteria aiming to appropriately control the complexity: (a) the values of coefficients were within the range of ± 5, and (b) the differences among the mean velocities of the generated waveform patterns of the three segments were no more than 1% when running the coefficients through the experiment setup. The waveform patterns of Seg1 and Seg3 were randomly generated and thus different for each trial, whereas the waveform patterns of Seg2 were repeated over trials for each participant. Twenty-four selected waveform patterns from a pool of more than three thousand generated patterns following the criteria mentioned previously were randomly assigned to each participant so that the repeated patterns in Seg2 differed for each participant.

### Procedure

**Figure 5:**
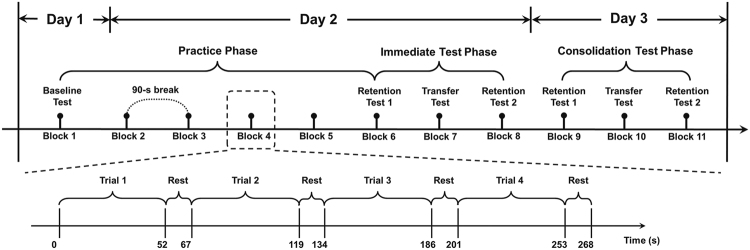
The schematic representation of the whole 3-day experiment.

Participants were told that they would see a small red dot (tracking target) occurring on the left middle of the screen and moving horizontally until reaching the right edge of the monitor. The task for all participants was to try their best to track the dot with the cursor as accurately as possible by controlling a stylus to draw on the tablet. For each participant, the whole experiment consisted of 11 blocks of the continuous tracking task on three days, denoted by Day 1, Day 2 and Day 3 respectively. On Day 1, Block 1 was taken as a tracking performance baseline test with randomly generated waveforms in Seg2. On Day 2, Blocks 2 to 6 and Block 8 had repeated waveform patterns in Seg2, while in Block 7, the waveforms in Seg2 were replaced by random patterns. Blocks 1 to 6 were considered as the practice phase and Blocks 6 to 8 were the immediate test phase, in which Block 6 and Block 8 were retention tests while Block 7 was a transfer test. On Day 3, another test phase of three blocks (i.e. Blocks 9 to 11) was performed in order to test the offline consolidation of implicit motor learning. To counterbalance the effect caused by the order of blocks, Block 9 and Block 11 were designed as retention tests while Block 10 was a transfer test, and the segment settings were the same as in the immediate test phase on Day 2. Each block was composed of four trials with a 15-s interval between two consecutive trials and a 90-s break was also provided between blocks. In order to get familiar with the continuous tracking task, participants completed a warm-up trial right before the formal task on each day. After completing the whole experiment, participants were first asked whether they had noticed anything particularly about the tracking waveform and then whether they had noticed any repetition of any part of the tracking waveform. The participants, who claimed that they had noticed the repetition, were further asked which part was repeated over trials. As a result, no participant reported any awareness of the repeated waveform pattern, which ensured that the waveform-specific learning was implicit. The schematic representation of the experiment process was shown in Fig. 5.

### Performance measures

In order to investigate the feasibility and sensitivity of the Pearson’s correlation coefficient on the vertical movement *r*_*v*_ for the IWS learning detection in the continuous tracking task paradigm, both *r*_*v*_ and RMSE were considered as the performance measures for comparison. The RMSE for each of the three segments was calculated respectively in each trial and then averaged across trials per block as the dependent measure of tracking performance in the corresponding block. Pearson’s correlation coefficient *r*_v_ for each segment in all trials was calculated as shown in Equation (2), where *X* = {*x*_1_, …, *x*_*n*_} represents the target vertical locations on the screen in time series, *Y* = {*y*_1_, …, *y*_*n*_} represents the cursor vertical locations on the screen in time series, $\mathop{x}\limits^{\bar{} }$ was the mean of *X*, and $\mathop{y}\limits^{\bar{} }$ was the mean of *Y*. For each of three segments, the average *r*_v_ across four trials per block was taken as the tracking performance in the corresponding block.

2 $${r}_{v}=\frac{{\sum }_{i=1}^{n}({x}_{i}-\bar{x})({y}_{i}-\bar{y})}{\sqrt{{\sum }_{i=1}^{n}{({x}_{i}-\bar{x})}^{2}}\sqrt{{\sum }_{i=1}^{n}{({y}_{i}-\bar{y})}^{2}}}$$
